# Clinical characteristics, treatment, and outcomes for elderly patients in a dedicated Covid-19 ward at a primary health care facility in western Norway: a retrospective observational study

**DOI:** 10.1186/s12913-024-11539-2

**Published:** 2024-09-19

**Authors:** Bård Reiakvam Kittang, Ane Tveiten Øien, Einar Engtrø, Marian Skjellanger, Kjell Krüger

**Affiliations:** 1 Department of Nursing Home Medicine, Municipality of Bergen, Bergen, Norway; 2 Department of Medicine, Haraldsplass Deaconess Hospital, Bergen, Norway; 3 Department of Clinical Science, University of Bergen, Bergen, Norway

**Keywords:** Covid-19, Nursing home, Advance care planning, Palliative care, Clinical Frailty Scale

## Abstract

**Background:**

The coronavirus pandemic has hit the oldest and frailest individuals hard, particularly patients and residents in nursing homes. In March 2020, we established a Covid-19 ward at a nursing home in Bergen, western Norway for elderly patients with Sars-CoV-2 infection and in the need of treatment and care in a primary health care facility. The aims of this study were to describe the organization of the ward, the clinical outcomes of infection, treatment, mortality rates in the population, the level of advanced care planning, and end-of-life care for those who died.

**Methods:**

We present patient characteristics, outcomes, vaccination status, treatment, decisions regarding treatment intensity upon clinical deterioration, and mortality for the patients in the ward. Clinical factors possibly related to a fatal outcome were analysed with chi square test (categorical variables) or t-test (continuous variables).

**Results:**

257 patients were included from March 2020 to April 2022. Fifty-nine patients (23.0%) developed respiratory failure. Ten patients (3.9%) were admitted to hospital. Advance care planning was undertaken for 245 (95.3%) of the patients. 30-day mortality rate decreased from 42 to 4% during the study period. Of the 29 (11.3%) patients who died, all were well alleviated in the terminal phase, and 26 (89.7%) of them had a Clinical Frailty Scale (CFS) value ≥ 7. A high score for CFS, respiratory failure and respiratory co-infection were significantly associated with Covid-19 related death within 30 days.

**Conclusions:**

Covid-19-related mortality markedly decreased during the study period, and a high score for CFS was related to a fatal outcome. Thorough planning of treatment intensity upon deterioration, low hospitalization rates, and good relief for those who died suggest that dedicated Covid-19 wards in nursing homes can provide good treatment for the patients and relieve other nursing homes and specialist health care services.

## Introduction

The burden of Covid-19 in nursing homes has been high throughout the pandemic, even after the introduction of Covid-19 vaccines [1]. Nursing home patients are susceptible to a severe course of respiratory infections and have a high prevalence of cognitive impairment [2, 3]. Along with resident crowding and reduced staffing capacity at nursing homes with Covid-19 outbreaks, this can hinder the implementation of adequate infection control measures, facilitate transmission of SARS-CoV-2 and increase Covid-19 related morbidity and mortality in this frail population [4–6]. As a consequence, particularly in the early phase of the pandemic, large outbreaks of Covid-19 with substantial mortality rates were observed in nursing homes [7, 8], and the nursing workload in both nursing homes and hospitals has been reported to be high [9, 10]. As such, advance care planning is an important aspect of proper care for vulnerable nursing home patients [11], particularly during the coronavirus pandemic.

In the public and private nursing homes in the municipality of Bergen in western Norway, nurse and doctor staffing is highly variable, and we experienced severe outbreaks of Covid-19 in three of our institutions early in the first pandemic phase [12]. Therefore, we established a citywide Covid-19 ward at Fyllingsdalen Treatment Centre in March 2020, with the purpose of providing close follow-up for particularly challenging nursing home patients with Covid-19 and relieving other nursing homes and hospitals in our community.

The aims of this retrospective, observational study was to describe the organization of the ward, and explore the clinical course of infection, treatment, mortality rates at the ward through the study period, clinical factors possibly related to a poor outcome, the level of advanced care planning, and the end-of-life care for those who died.

## Materials and methods

### Organization of the ward and study population

The municipality of Bergen has 23 public and 11 private nursing homes, with approximately 2000 long-term beds and 500 short-time beds. The Covid-19 ward accounted for 11 out of 96 short-term beds at Fyllingsdalen Treatment Centre (FTC). The admission criteria were not strict, but patients with a RT-PCR verified Sars-CoV-2 infection fulfilling one or more of the following criteria were prioritized for admittance: (i) long- or short-term residents at other nursing homes requiring increased staffing and/or increased medical support due to cognitive failure and/or a severe clinical course, (ii) patients stabilized at hospital, but still requiring institutional care, and (iii) old and frail individuals not able to cope at home/care home services due to covid-19. During the establishment period of the ward, information regarding admission criteria was conveyed to nursing homes in the municipality of Bergen and the two local hospitals: Haukeland University Hospital (HUH) and Haraldsplass Deaconess Hospital (HDH).

The medical activity at the ward was closely monitored and supervised by an infectious disease specialist. Dedicated nurses and doctors received special training in infection control measures, with a particular focus on proper use of personal protective equipment (PPE), including eye and face protection, gloves, and single use gowns, along with correct collection and handling of clinical samples and waste management. Since the number of patients admitted to the ward varied considerably during the study period, there was a high degree of flexibility of ward staffing. In time periods with particularly challenging patients and/or high turnover rates, nurse and doctor resources were allocated from other wards at FTC. Furthermore, the ward was prioritized regarding access PPE and O2-concentrators. Since many patients had dementia and a need to wander around, the ward consisted of separate rooms, a spacious hall, and a large living room. One or two nurses was attending the cohort area at regular shifts. The ward doctor had several rounds with patients visits each weekday and had daily contact with the supervising infectious disease specialist. During weekends the attending nurses consulted a nursing home doctor on call in the municipality of Bergen when needed.

Altogether, 261 patients with Covid-19 were treated in the ward. These patients were transferred either from HUH or HDH, other nursing homes, care home services or their private homes. The patients admitted from other nursing homes were from 21 different institutions in the municipality of Bergen, and these residents all had a severe disease course and/or dementia preventing proper isolation during infection. During their stay at the ward, the patients were systematically evaluated regarding frailty and treatment intensity upon clinical deterioration, including cardiopulmonary resuscitation status, potential indications for admission to hospital, systemic corticosteroid treatment upon Covid-19 related respiratory failure, and antibiotic treatment for respiratory co-infections. Furthermore, morphine and midazolam were ordinated as emergency medication for all patients upon admission, in case of the development of an acute respiratory crisis.

### Diagnosis and clinical assessment

Patients were included from March 2020 to April 2022, when the ward was closed. All were diagnosed with Covid-19 using RT-PCR for SARS-CoV-2 from nasopharyngeal or throat swabs [13]. Clinical information was obtained from the nursing home’s semi-structured medical records system. Frailty assessment was conducted using the Clinical Frailty Scale (CFS), ranging from 1 (very fit) to 9 (terminally ill), classifying patients as non-frail (1–4), moderately frail (5–6), or severely frail (7–9) [14]. The assessment of the value for CSF was based on the degree of frailty at least two weeks prior to Covid-19, was conducted by the ward doctors based on information in medical records, from the patients themselves, and/or next of kin. Respiratory failure was defined as persistent hypoxia (oxygen saturation < 90% without supplementation of oxygen in lung-healthy individuals, or oxygen saturation < 85% without supplementation of oxygen in patients with chronic lung disease). Data on the quality of palliative care for those who died of Covid-19 was based on detailed documentation in the medical records system, from both nurses and doctors working in the unit, by using a structured journal scheme called “Livets Siste Dager” (“The Last Days of Life”), which is regularly used in Norwegian hospitals and primary care facilities, and developed and refined from Liverpool Care Pathway [15]. Covid-19 related death was defined as death within 30 days after symptom onset, or from the time of positive test for initially asymptomatic patients.

### Statistical analysis

Categorical variables (gender, respiratory failure, and respiratory co-infection) were analysed by chi square test. Continuous variables (mean age and mean score for CFS) were analysed by t-test. A two-sided p-value < 0.05 was considered statistically significant. All analyses were performed by using JMP 16.2.0 (SAS Institute Inc.). Vaccination status, along with treatment with corticosteroids and anticoagulants were omitted from the statistical analysis of risk factors for death, as these prophylactic and treatment measures were not available during the first pandemic phase, when most of the deaths occurred.

## Results

We included 257 out of a total of 261 patients admitted to the ward. Four patients opted out. Eighty-five patients (33.1%) were transferred from hospital, while the remaining 172 (66.9%) patients were admitted from other nursing homes (*n* = 149, 58.0%), care home services (*n* = 10, 3.9%), or their own homes (*n* = 13, 5.1%). Ten patients (3.9%) were acutely admitted to a hospital from the ward, all with severe Covid-19 related respiratory failure or clinical signs of septicaemia caused by respiratory co- infection.

**Fig. 1:**
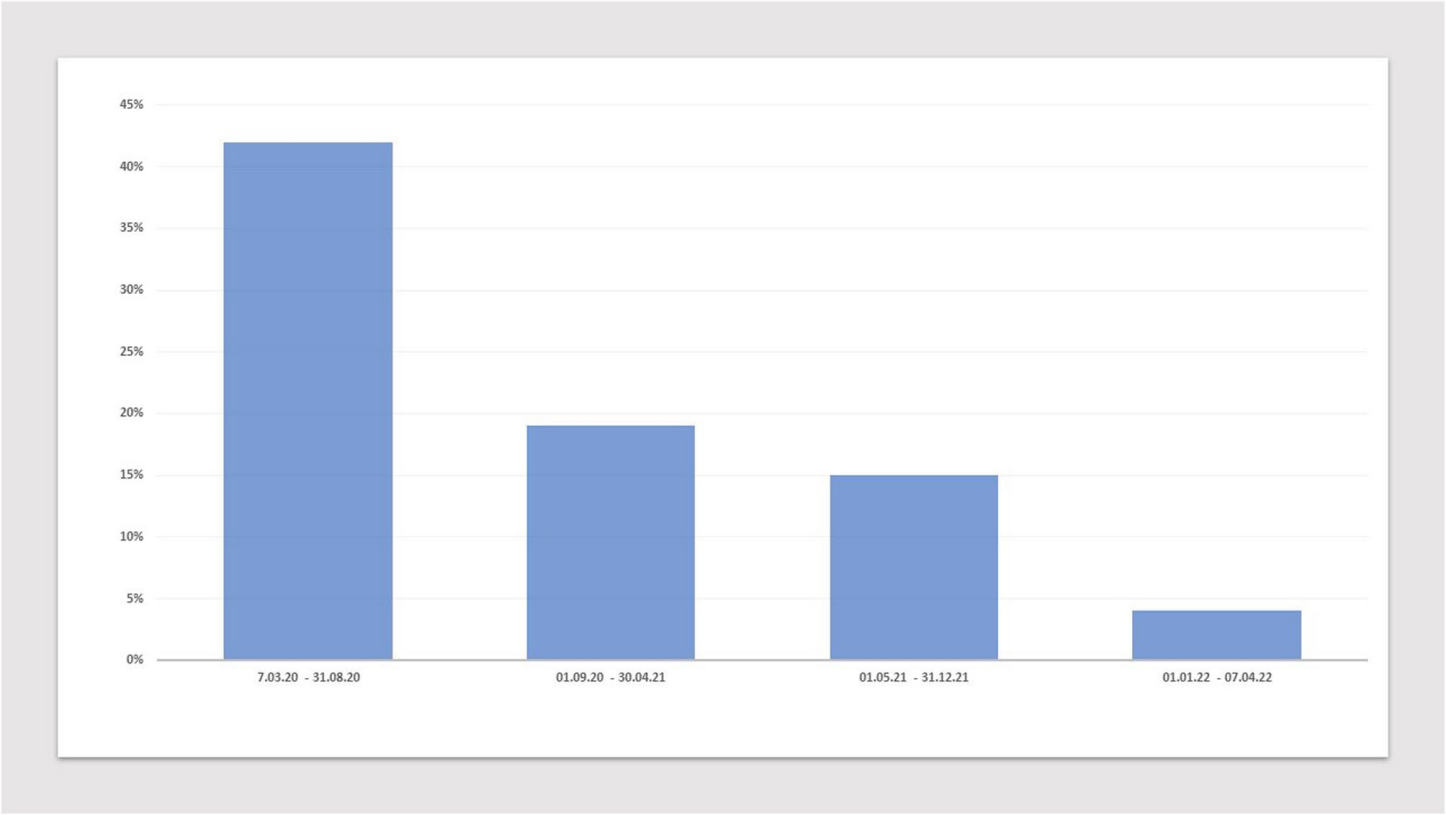
Covid-19 related mortality rates at Fyllingsdalen Treatment Centre during March 2020-April 2022 Covid-19 related 30-day mortality rates at the ward during the study period from March 2020 to April 2022. Mortality rates (%) are displayed on the y-axis, and different time periods on the x-axis. The data are separated into four time periods: i) The first pandemic phase, with presumably Wuhan – 1 as the dominating Sars-CoV-2 variant. ii), the pandemic phase with the introduction of anticoagulation therapy for most patients with covid-19, and corticosteroid therapy for selected patients with respiratory failure. In this period, there was presumably a steadily transition of the dominating variant of Sars-Cov-2 from Wuhan -1, to Alpha, and then to Beta. iii), the pandemic phase with Delta presumably as the dominating variant of Sars-Cov-2. iiii), The pandemic phase with Omicron presumably as the dominating variant of Sars-Cov-2 The total number of patients in the four groups, from i), ii), iii) and iiii), were 19, 28, 56 and 154, respectively

We observed a significant reduction in mortality throughout the study period; from 42% in spring 2020 to 4% from January to April 2022 (Fig. 1).

As shown in Table 1, the mean age and score for CFS were 83.2 years and 6.3 respectively. Women accounted for 166 (64.6%) of the patients. Fifty-nine patients (23.0%) developed respiratory failure, of which 33 (55.9%) received treatment with corticosteroids. Advance care planning was performed for 245 (95.3%) patients, and all of 29 patients who died during the course of infection were well alleviated in the terminal phase. Of those who died, 26 (89.7%) had a CFS score > = 7.

**Table 1:** Clinical characteristics, advance care planning and palliative care for 257 patients at the Covid-19 ward at Fyllingsdalen Treatment Centre during March 2020-April 2022

Variable	Result
Age (years), mean (median, range)	83.2 (85, 42–99)
Female gender, n (%)	166 (64.6)
Score for Clinical Frailty Scale mean (median, range)	6.3 (7, 3–9)
Covid-19 related respiratory failure, n (%)	59 (23.0)
Fatal outcome, n (%)	29 (11.3)
Advance care planning performed (%)	245 (95.3)
Adequate palliative care in the terminal phase for those who suffered from Covid-19 related death (%)	29 (100)

As displayed in Table 2, patients who suffered from Covid-19 related death had a higher mean CFS score (*p* < 0.001), more often respiratory failure (*p* < 0.001) and respiratory co-infection treated with antibacterial agents (*p* = 0.0340) than those who survived. One hundred and eighty-four (71.6%) of the patients were vaccinated against Covid-19 with one or more doses, whereas 33 (12.8%) and 182 (70.8%) of the patients received treatment with corticosteroids and anticoagulants, respectively.

**Table 2:** Clinical characteristics, vaccination status and treatment among 257 patients in the Covid-19 ward at Fyllingsdalen Treatment Centre during March 2020-April 2022

Variable	Dead patients (*n* = 29)	Surviving patients (*n* = 228)	*p*-value*
*Patient characteristics*			
Age (years), mean (median, range)	85.2 (87, 69–98)	82.9 (85, 42–99)	0.4351
Female gender, n (%)	18 (62.1)	152 (65.5)	0.7630
Score for Clinical Frailty Scale, mean (median, range)	7.3 (7, 5–9)	6,3 (6, 3–8)	< 0.0001
Covid-19 related respiratory failure, n (%)	19 (65.5)	23 (10.1)	< 0.0001
Respiratory co-infection treated with antibacterial agents, n (%)	7 (24.1)	24 (10.5)	0.0340
*Vaccination/treatment*,* n (%)*			
Vaccinated **, n (%)	9 (31.0)	175 (76.8)	
Corticosteroid treatment of Covid-19 related respiratory failure***, n (%)	6 (20.7)	26 (11.4)	
Anticoagulant therapy****, n (%)	17 (58.6)	165 (72.3)	

*Analysed with t-test (continuous variables) or chi square test (categorical variables), level of significance, *p* < 0.05

** Vaccinated against Covid-19 with one or more doses

*** Prednisolone 40 mg orally q.d for 7–10 days, or dexamethasone 6 mg orally q.d for 7–10 days

**** Enoxaparin 4000 IE subcutaneously q.d, or continuation of treatment with direct oral anticoagulants

## Discussion

To our knowledge, this is the first study describing clinical patient characteristics, structured advance care planning and quality of palliative care in a dedicated Covid-19 nursing home ward. During the study period, we observed a significant decrease in Covid-19 associated mortality rates, from 42% in the early pandemic phase to 4% in the Omicron era. Similar findings were evident from international studies [16, 17] and probably related to the introduction of Covid-19 vaccines, the emergence of Sars-Cov-2 variants (particularly Omicron) with attenuated pathogenicity, and perhaps, the introduction of novel treatment (anticoagulants and corticosteroids) in our Covid-19 ward from the second pandemic phase. Up until Covid-19 vaccination was initiated at our nursing homes in January 2021, the 30-day mortality rate in our patient population was approximately 25%, comparable to those observed in other countries [7, 18]. 

Coronavirus vaccination protects against severe outcomes of Covid-19, also among the elderly in and outside of nursing homes [19, 20], and a high chronological age is associated with poor outcomes [21]. In our sample, the mean age was, as expected, high among both those who died, and those who survived the infection, and chronological age was not associated with fatal outcome. Furthermore, the vast majority of those who died had a CFS score that indicated severe frailty, and a high CFS score was significantly associated with death. Hence, in nursing homes, assessment of clinical frailty appears to be more important than chronological age when evaluating the risk for severe Covid-19 outcomes, in line with findings from studies on Covid-19 patients in hospital and Spanish nursing homes [22–24].

Treatment with systemic corticosteroids of hospitalized Covid-19 patients with respiratory failure has been shown to reduce mortality [25]. However, to our knowledge, no data on the effect of such treatment for nursing home patients with Covid-19 has been published. Given the relatively high mortality and incidence of severe respiratory failure among our patients in the early stages of the pandemic [12], along with the thrombogenic properties of SARS-CoV-2 and a high incidence of venous thrombotic events among nursing home residents in general [26, 27], we chose to offer anticoagulants to a majority of the patients, along with corticosteroid treatment to more than half of those with Covid-19 related respiratory failure from September 2020 until April 2022.

Important aspects in the care for Covid-19 patients in nursing homes include a systematic assessment of proper treatment intensity for each patient, and providing good palliative care for those who die from the infection [28, 29]. In our material, advance care planning was undertaken for more than 90% of the patients. Only 10 patients were admitted to hospital during the infection, and all 29 who died were well palliated in their final days of life according to structured information from their medical records. Hence, our findings suggest that dedicated Covid-19 wards in nursing homes offer good care for the patients and provide relief for other nursing homes and hospitals.

This study has several limitations. First, retrospective collection of clinical information and the inclusion of a relatively small number of patients from a rather limited geographical area, in a high-income country with a resourceful health care system [30], may have limited the transferability of our findings to other regions/countries. Second, we did not include data on comorbidities potentially contributing to a poor outcome. Nevertheless, the use of structured journal forms and CFS likely contributed to increasing the reliability and validity of our data. Furthermore, CFS score probably reflects the burden of comorbidities to a certain extent. Third, the good and flexible access to clinical personnel and PPE might have influenced both outcomes and quality of care in the Covid-19 ward compared to other nursing home wards. Another inherent limitation introducing biases in the interpretation of our data, was the development of Covid-19 vaccines, the steadily changing virus variants, and the changes in treatment options during the study period. As such, statistical analyses of independent risk factors for fatal outcome were not performed.

## Conclusions

We found a significant reduction in Covid-19 related mortality during the study period. The hospital admission rate was low, palliative care in the final days of life was good for those who died, decisions regarding proper treatment intensity were available for the vast majority of the patients, and very few of them were acutely admitted to hospital. Through our experience from this Covid-19 ward, we have learnt that a systematic approach towards the patients, along with allocation of PPE and human resources might ease the burden of pandemics on both primary care institutions and hospital, and possibly lead to better patient care.

Hopefully, our approach towards care elderly patients during the Covid-19 pandemic, might inspire future health care plans for this frail population if a new pandemic were to occur in the years to come.

## Data Availability

The datasets used and/or analysed during the current study are available from the corresponding author on reasonable request.
